# Clinic at the Mercy Hospital, by Prof. N. S. Davis

**Published:** 1858-10

**Authors:** E. A. Steele


### CLINIC AT THE MERCY HOSPITAL, MONDAY, OCTOBER 18TH, BY PROF. N. S. DAVIS.

REPORTED BY E. A. STEELE, A.B.

GENTLEMEN,—This morning I desire to call your attention to the interesting features of the case now before you.

On examining the patient this morning you will find the pulse moderately increased in frequency; the skin dry, and universally of a deep yellow color; the tongue covered with a thin yellowish fur, but not dry; the epigastrium full and tender to pressure, though the effect of pressure is more a feeling of sickness or nausea than of acute tenderness. The same feeling of undue fulness and nausea is felt a little while after taking food or drink. The bowels are inactive, and the patient drowsy. The urine is scanty and of a deep yellowish brown color, from the coloring matter of the bile. The patient tells me he has been laboring under these symptoms during the last eight or ten days, and has taken some cathartic medicine. In one sense, the diagnosis in this case is very easy. The deep yellow hue of the skin, the conjunctiva, and even the secretions, would lead any of you to say he had the *jaundice*. But I wish to impress strongly on your minds the fact, that *jaundice* is not a disease; that the word itself conveys no idea of a definite pathological condition. On the contrary, it, like *dropsy*, is a mere symptom, which may be produced by a variety of pathological conditions.

Obstruction of the biliary ducts by calculous concretions, too great viscidity of the bile itself, atony or want of action in the capillary branches of the biliary ducts in the liver, inflammation and swelling of the lining membrane of the ducts, or of the mucous lining of the duodenum; in a word, everything that is capable of retarding or arresting the free flow of bile from the secreting lobules of the liver into the duodenum, is capable of producing *jaundice*. Hence, in a case like this, a proper diagnosis consists, not in pronouncing the disease to be jaundice, which really means nothing more than a saffron color of the cutaneous surface from excess of retained bile, but in determining carefully which of the several pathological conditions capable of interfering with the proper exit of bile from the liver, actually exists in the patient before us.

You can only distinguish these several pathological conditions by clearly appreciating the symptoms and ascertaining the history of your patient. As long as the biliary calculi remain within the gall bladder, little or no phenomena result from their presence; but when they, in their egress, are passing along the cystic or common duct, you will have a train of phenomena very different from that which characterizes the jaundice, depending upon the inflammation of the duodenum. The patient is suddenly attacked with intense pain in the epigastric and hypochondriac regions; the stomach sympathizes, and we have nausea, cardialgia, and sometimes vomiting. The abdominal muscles are thrown into spasmodic contraction; the extremities become cold; the body bathed in perspiration, and the pulse is often hard and contracted, but seldom accelerated. The pain is more intense than that which attends any form of inflammation, yet the pulse, as a general thing, is but little disturbed either in force or frequency. If the obstruction is not removed, the urine becomes highly colored, and in most cases the patient grows brightly jaundiced. This will not always be the case, since, while the concretion is in the cystic duct, there need be no jaundice; and if, from its angular shape, it only partially obstructs the common duct, it may be only transient, appearing and disappearing with rapidity. But, in some cases, you will find that the efforts of nature to relieve the obstruction will be unavailing; it remains impacted in the duct, and its presence excites inflammation; lymph is thrown out, and the canal is entirely closed. In other cases, these concretions have been found to make their way into the duodenum, by ulceration, and were thus discharged. But that which arises from the impaired condition of the absorbent ducts is more difficult of diagnosis: there is a weight and heaviness in the right side, sometimes enlargement of the liver, but the patient becomes gradually jaundiced, and presents no very positive symptoms. On the contrary, that, which (being satisfied as to the pathology) I have styled the "*hepatico duodenitis*," is easily distinguished from the former. The patient does not have his usual appetite; in some, a slight tenderness over the epigastrium, with an unpleasant fulness upon taking food. The unpleasant symptoms pass away, and the patient neglects to apply for relief. But in their recurrence, perhaps aggravated by delay, conceives the idea that this fulness and sense of nausea may be effectually relieved by an emetic, or, in order to arouse the torpid condition of the liver, a cathartic is taken. The emetic does not produce the relief anticipated; very little bile is discharged, and only a thin mucus secretion is thrown off the stomach. In more severe cases, you will have all these symptoms aggravated; the epigastrium becomes full and tender on the slightest pressure, and whatever is taken into the stomach is rejected immediately. There is deficient secretion generally; skin dry and hot; pupil of the eye dilated, and you have the usual symptoms that characterize the typhoid condition. No delirium, but patient becomes dull and drowsy from the narcotic effects of the natural coloring matter of the bile, together with the accumulation of urea in the circulation from suspended secretion. Sometimes vomiting will occur of a dark coffee-ground serous fluid, analogous to the black vomit of yellow fever. This will not depend upon the quantity of fluid taken, but the serum of the blood, with degenerated red corpuscles, is effused through the capillaries of the mucous membrane, and changed in its color by the acid of the stomach. If these symptoms continue, the skin assumes a more bronzed hue, pulse grows small, and the patient dies.

I have seen but two fatal cases of this form of disease, and in one of them a post-mortem examination was made. I found the whole surface of the mucous membrane of the duodenum strongly injected with blood; several spots of a dark brown color, and decidedly softened; with general tumefaction or swelling of the membrane. The same marks of inflammation existed in the lining of the hepatic ducts throughout their whole extent; and the liver was moderately enlarged, the bloodvessels of its central portion much engorged, and the texture of the same part so softened as to be easily lacerated with the finger. The other organs of the body gave no important marks of disease.

This examination fully confirmed the opinions I had before entertained, viz: that the cases of acute jaundice which we so frequently meet with, especially during protracted wet weather in the autumn, and sometimes in the spring; and which, like the patient before us, present a sense of fulness, tenderness and nausea in the epigastrium, with slight febrile symptoms and general lassitude,—are simply cases of a low grade of inflammation in the mucous membrane of the duodenum and hepatic ducts. The increased vascularity and consequent tumefaction of the membrane, obstructs more or less the flow of bile through the ducts, causing much of it to be reabsorbed and diffused throughout the tissues, and giving to the whole surface the deep yellow color which is so prominent a feature of the case before us. Being satisfied in reference to the diagnosis and pathology of the case, the next inquiry is in regard to the indications for treatment. These are the same as in any other inflammation of a sub-acute character; namely, to lesson the sensitiveness and vascularity of the inflamed structure, and to promote secretion generally, which has been diminished by the depressing effect of an excess of bile in the blood. In severe cases, leeches to the epigastrium, followed by fomentations and subsequently a blister, will be of much service. But in cases like the one before us, a judicious combination of anodyne and alterative medicine, with an occasional laxative, will speedily restore the patient. Many regarding chiefly the torpid condition of the bowels, the sense of fulness in the epigastrium, and the yellowness of the skin, have commenced the treatment of these cases with an active emetic, followed by mercurial cathartics. But I have never known a case of this kind in which the operation of an emetic was not injurious. The remedies that have generally succeeded best in my hands are the following:

| | | |
|---|---|---|
| ℞ | Pulv. Doveri, . . . | 30 grs. |
| | Pulv. nit. potassa, . . | 30 " |
| | Sub-murias hydrarg., . . | 10 " |

Mix; divide into six powders, and give one every four hours until all are taken. In some cases the stomach is so irritable, that the pulv. Doveri will be rejected by vomiting. In such, we may give, as a substitute, from $\frac{1}{8}$ to $\frac{1}{4}$ of a grain of morphine in each powder. If no evacuation of the bowels should occur, after the exhibition of the remedies in the quantities indicated, I would give a laxative of a mild character; the Seidlitz powder is to be preferred; being both alkaline and saline in its constituents, it will serve to render the bile less viscid, and promote the action of the kidneys.. If no special effect should follow from the use of two or three of these powders, I would not persist in their administration, but suspend all internal remedies, and order an enema of salt and water. After the free movement of the bowels in this way, I would return to the powders of nitras potassa and pulv. Dover's, leaving out the mercury altogether, or reducing the proportion just as the discharges from the alimentary canal and the general condition of the patient might indicate. I have hardly ever found that when the bowels were opened, after a repetition of the medicine the second time, that full and general secretion was not established, and the symptoms of the disease rapidly ameliorated.

This patient, on his admission to the Hospital two days since, was put upon the use of the remedies just indicated, and his bowels have been moved for the first time this morning. Although a decided improvement has taken place, especially in reference to the nausea and distress in the epigastrium, yet the alvine evacuations were light colored, and the skin continues dry and above the natural temperature.

We will, therefore, direct a continuance of the pulv. Doveri and nit. potassa, 5 grs. each, with calomel 1 gr., repeated every four hours until six additional doses have been taken. Then we will cause a movement of the bowels by the same means as before. On some future morning your attention will again be called to the patient, when you will learn the results of the treatment.

Three mornings subsequently, the Doctor again called the attention of the class to this patient, and remarked that after the completion of the treatment directed at the previous interview, two or three free dark green evacuations were procured, showing that the bile flowed freely through the ducts; and at the same time the tenderness, nausea and the febrile symptoms were entirely removed. The skin was moist and the urine copious, but like the skin highly impregnated with the coloring matter of bile. To keep up a free state of the secretions until the blood should be fully depurated, and at the same time restore a healthy tone to the digestive organs, the following pill had been ordered:

| | | |
|---|---|---|
| ℞ | Sulphas ferri, . . . | 20 grs. |
| | Pulv. aloes, . . . | 10 " |
| | Ext. hyosciamus, . . . | 20 " |

Mix, and divide into twenty pills. One had been taken before each meal, and 2 grs. of blue mass at bedtime. The patient now feels quite well, and is able to take food without any inconvenience.

The yellow color of the conjunctiva and skin is also much diminished. The Doctor remarked that the blue mass might be omitted after one or two more doses, and only the tonic-laxative pill continued. He also remarked that such patients, when discharged, should be instructed to wear flannel next the skin, to avoid rich and highly seasoned food, and all stimulating drinks, as the best mode of preventing a relapse.

The patient was discharged quite well on the ninth day after admission.

